# Habitat heterogeneity, temperature, and primary productivity drive elevational gradients in avian species diversity

**DOI:** 10.1002/ece3.7341

**Published:** 2021-05-01

**Authors:** Kristen G. Dillon, Courtney J. Conway

**Affiliations:** ^1^ Idaho Cooperative Fish and Wildlife Research Unit Department of Fish and Wildlife Sciences, University of Idaho Moscow ID USA; ^2^ U. S. Geological Survey Idaho Cooperative Fish and Wildlife Research Unit Department of Fish and Wildlife Sciences, University of Idaho Moscow ID USA; ^3^ Present address: U.S. Bureau of Reclamation Denver CO USA

**Keywords:** altitudinal gradient, altitudinal zonation, biodiversity, elevational gradient, habitat heterogeneity, montane ecosystems, primary productivity, species diversity, temperature

## Abstract

**Aim:**

Anticipating and mitigating the impacts of climate change on species diversity in montane ecosystems requires a mechanistic understanding of drivers of current patterns of diversity. We documented the shape of elevational gradients in avian species richness in North America and tested a suite of a priori predictions for each of five mechanistic hypotheses to explain those patterns.

**Location:**

United States

**Methods:**

We used predicted occupancy maps generated from species distribution models for each of 646 breeding birds to document elevational patterns in avian species richness across the six largest U.S. mountain ranges. We used spatially explicit biotic and abiotic data to test five mechanistic hypotheses proposed to explain geographic variation in species richness.

**Results:**

Elevational gradients in avian species richness followed a consistent pattern of *low elevation plateau‐mid‐elevation peak* (as per McCain, 2009). We found support for three of the five hypotheses to explain the underlying cause of this pattern: the habitat heterogeneity, temperature, and primary productivity hypotheses.

**Main Conclusions:**

Species richness typically decreases with elevation, but the primary cause and precise shape of the relationship remain topics of debate. We used a novel approach to study the richness‐elevation relationship and our results are unique in that they show a consistent relationship between species richness and elevation among 6 mountain ranges, and universal support for three hypotheses proposed to explain the underlying cause of the observed relationship. Taken together, these results suggest that elevational variation in food availability may be the ecological process that best explains elevational gradients in avian species richness in North America. Although much attention has focused on the role of abiotic factors, particularly temperature, in limiting species’ ranges, our results offer compelling evidence that other processes also influence (and may better explain) elevational gradients in species richness.

## Graphical abstract

We documented the shape of elevational gradients in avian species richness across the entirety of six U.S. mountain ranges and tested mechanistic hypotheses to explain those patterns. Elevational gradients in avian species richness followed a consistent pattern of low elevation plateau‐mid‐elevation peak (as per McCain, 2009). We found support for three hypotheses to explain this pattern: the Habitat Heterogeneity, Temperature, and Productivity Hypotheses.

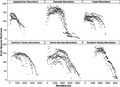

## 1 INTRODUCTION

The primary goal of many contemporary conservation efforts is to maintain species diversity in the face of a warming climate (Heller & Zavaleta, 2009). Montane ecosystems are unique in that they are expected to provide thermal refugia for species displaced by a changing climate, while simultaneously supporting habitat types considered most at risk of disappearing due to global warming. Many recent studies have documented shifts in species distributions in response to warming (Perry et al., 2005; Gillings, Balmer, & Fuller, 2015) and these shifts will likely change how species are distributed along elevational gradients within montane systems as species are displaced from their current elevational range. However, understanding the impacts of changing climate conditions on species distributions and the corresponding changes in elevational patterns in species richness requires a scientific understanding of the baseline shape of those patterns. Moreover, species’ ability to move up in elevation as climates warm will likely be constrained by ecological processes other than simply thermal tolerance that limit species’ distributions, such as food resource availability, suitable breeding habitat availability, predation risk, etc. Hence, our ability to predict and potentially manage the impacts of climate change requires understanding the ecological processes driving elevational clines in diversity (Newton, 1980; Martin, 1988a; Graham et al., 2014). Although there are many existing studies of the mechanistic drivers of elevational clines in species diversity, there is still a lack of consensus on the shape and causes of elevational patterns of species richness, such that different relationships have been reported among regions, taxa, and scales of analysis (Rahbek, 1995; McCain, 2009; Quintero & Jetz, 2018). We took a novel analytical approach to document the relationship between elevation and species richness of breeding birds in North America and to test five common mechanistic hypotheses to explain the underlying cause(s) of that relationship.

**FIGURE 1:**
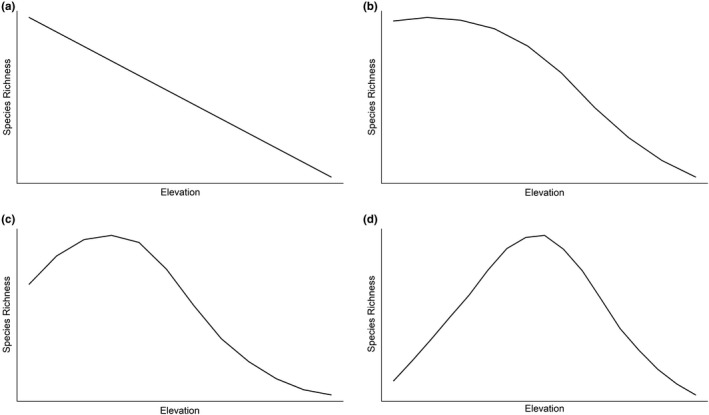
Conceptual graphs of the four different relationships between species richness and elevation that have been described or reported in past studies (Rahbek 1997, McCain 2009): a) linear elevational decline, b) low elevation plateau, c) low elevation plateau with mid‐elevation peak, and d) mid‐elevation peak

How does species richness of animals vary across elevational gradients? There is considerable ambiguity but four general patterns of elevational variation in species richness reported in past studies were described, quantified, and named by McCain (2009): richness declines linearly with elevation, a low elevation plateau, a low elevation plateau with a mid‐elevation peak, and a mid‐elevation peak (i.e., mid‐domain effect) (Fig. 1). Documenting the shape of the relationship between species richness and elevation through replication across mountain ranges and spanning their entire elevational extent is an important prerequisite to understanding the primary causes of elevational clines in diversity. Documenting the relationship between species richness and elevation and the extent to which the relationship varies among mountain ranges can help guide efforts to determine the underlying cause of elevational gradients in species richness.

Many of the hypotheses that have been proposed to explain elevational variation in species richness are derived from hypotheses to explain geographic variation in biodiversity in general. These hypotheses strive to explain variation in species richness as a function of biotic factors (e.g., competition, habitat heterogeneity, primary productivity), abiotic variables (e.g., temperature, precipitation), spatial factors (e.g., species–area relationships (SAR), mid‐domain effects (MDE)), or evolutionary history (Connor & McCoy, 1979; Huston, 1979; Rahbek, 1997; McCain, 2009; Pan et al., 2016). The hypotheses are not all mutually exclusive and testing among them has proven difficult due to the inherently broad spatial scope of the patterns, the different scales of organization among the hypotheses, and correlations among drivers underlying the existing hypotheses. Indeed, a pattern as broad as geographical variation in species diversity is potentially influenced by complex interactions of multiple ecological and evolutionary processes. Hence, we need to take a continental or global approach and test these alternative hypotheses simultaneously (McCain, 2009; McCain, 2010; Szewczyk & McCain, 2016; Quintero & Jetz, 2018). The five most‐commonly‐invoked hypotheses to explain geographic variation in biodiversity include the habitat heterogeneity hypothesis, the temperature hypothesis, the precipitation hypothesis, the primary productivity hypothesis, and the species–area relationship hypothesis. Our novel approach to quantifying elevational variation in avian species richness in North America also allowed us to explicitly test these five commonly proposed hypotheses to explain elevational gradients in avian species richness.

We used high‐resolution, biologically informed geospatial maps derived from species distribution models to calculate species richness in the six largest mountain ranges in the conterminous United States. This dataset, which enabled us to sample the entirety of multiple mountain ranges, allowed us to calculate species richness without the biases associated with uneven sampling effort across elevational gradients or limited study scale that other approaches have had to confront (Nogues‐Bravo, Abraujo, Romdal, & Rahbek, 2008; McCain & Grytnes, 2010). Previous studies have reported all four major patterns of elevational variation in species richness in birds (Rahbek, 2005; McCain, 2009), but no single ubiquitous pattern or explanation has emerged. Understanding the underlying cause(s) of elevational gradients in species richness will help better predict and mitigate the effects of climate change on species diversity. By compiling broad‐scale geospatial data, we were able to sample entire mountain ranges from base to peak and our sample units were not constrained by topography as they might be in field‐based sampling. Additionally, we measured species richness across each of six mountain ranges in their entirety, rather than along isolated transects. Our objectives were to rigorously describe the relationship between elevation and avian species richness in the United States and to explicitly test five of the leading hypotheses to explain that elevational gradient: Habitat heterogeneity, primary productivity, precipitation, temperature, and the species–area relationship.

## 2 METHODS

### 2.1 Species richness gradient

We used data from the six largest mountain ranges in the conterminous United States: the Coast, Cascade, Sierra Nevada, Northern Rocky, Southern Rocky, and Appalachian Mountains. We considered the Northern and Southern Rocky Mountains as separate ranges for analysis given that a large expanse of plains (across Wyoming and Idaho) separates the two. We used a topographic basemap in ArcMap software (ESRI, 2015) and a Digital Elevation Model (U.S. Geological Survey, 2018) to delineate the boundaries of each of the six mountain ranges. We divided each of the six mountain ranges along their crest into the east and west aspect, given that all six mountain ranges run north‐to‐south and their eastern and western aspects often experience very different climatic conditions. We then created 12‐18 transects in each mountain range that spanned from the base to the crest of the mountain range bounded by one degree of latitude on each side. We used sample units along each transect to calculate species richness and each sample unit was a polygon encompassing all area between two degrees of latitude and two 100m‐increment elevation contours (e.g., all area from 100m‐200 m.a.s.l. was one polygon, 200m‐300m was a second, etc.). To calculate species richness within each sample unit, we used ArcGIS 10.3 to overlay 30m resolution maps based on species distribution models from the U.S. Geological Survey GAP Analysis program (U.S. Geological Survey, 2018) onto our sample units and calculate the number of species present in each sample unit during the avian breeding season. We included all species with a “summer” or “year‐round” classification in the GAP dataset in the analysis (i.e., we excluded species that were only present in an area during winter or migration). The species distribution maps from the GAP Analysis program are based on output from species distribution models (SDMs) that were created specifically for each species in the conterminous United States. (U.S. Geological Survey, 2018; Gergely et al., 2019). The SDMs are quantitative deductive models that incorporate elevation and many other remote‐sensed habitat variables to predict habitat suitability, and the variables used in each species’ model are based on habitat relationships in the literature. Hence, the maps derived from the GAP program’s species distribution models are unique in that they are not the typical, coarse, two‐dimensional range maps smoothed across elevation and other important habitat variables. GAP distribution maps are created by applying a deductive habitat model to remote‐sensed data layers within a species’ range. A team of GAP program biologists constructed a separate deductive model for each species by compiling habitat characterizations from species accounts in peer‐reviewed literature and databases and including factors such as elevation, proximity to water, land use class, extent of human disturbance, hydrologic variables, patch size, extent of ecotones, etc. (Gergely et al., 2019). The maps derived from these models are fine‐grained, three‐dimensional distribution maps sensitive to species‐specific biotic and abiotic habitat requirements with 30‐m resolution (U.S. Geological Survey GAP Analysis Program, 2018; Gergely et al., 2019). We calculated avian species richness for each 100‐m sample unit by summing the number of species of breeding birds in each sample unit. We used these data to document the elevational gradient in avian species richness (i.e., how species richness changes with elevation).

### 2.2 Predictors of species richness

We used publicly available geospatial data sources to quantify the following environmental conditions at each sample unit: number of land cover types, mean productivity, mean precipitation, mean daily minimum temperature, and total area. We derived the area of each sample unit directly from the sample unit attribute table and the latitude of each sample unit from the ESRI World Latitude and Longitude Grids dataset (https://www.esri.com). We used the National Land Cover Database (NLCD) to document the land cover at each sample unit and calculated the number of distinct land cover types within each sample unit. The NLCD classifies all land cover into eight broad land cover categories (water, developed area, barren, forest, shrubland, herbaceous, cultivated, and wetland) and into many sub‐categories of land cover within each of the eight broad land cover categories (Homer et al., 2015). Land cover heterogeneity is widely thought to contribute to species diversity at the landscape scale (Tews et al., 2004). We calculated mean elevation for each sample unit from the GAP elevation dataset (U.S. Geological Survey, 2018). We used the Enhanced Vegetation Index (EVI) as an indicator of primary productivity and calculated mean EVI for each sample unit (NASA LP DAAC). Previous research has found that EVI is a robust estimate of primary productivity across ecosystem type (Shi et al., 2017). Finally, we calculated mean daily minimum temperature and mean precipitation for each sample unit from PRISM datasets (Prism Climate Group, 2015). The landcover data used to inform the species distribution models was collected in 2001. To synchronize the timeframe of the data used in this analysis, all other time‐dependent data (e.g., climatic variables, landcover data) was also collected for 2001. Moreover, this analysis examined species richness during the avian breeding season, defined as March through August, and so we used these same months as the basis for calculating the other time‐dependent data (i.e., temperature, precipitation, EVI).

### 2.3 Statistical analysis

We used a negative binomial regression to analyze the shape of the relationship between avian species richness and elevation, after controlling for latitude (see rationale below) (Venables & Ripley, 2002; R Core Team, 2018). We selected a negative binomial model rather than a Poisson regression due to overdispersion of the response variable. Once we described the relationship between species richness and elevation, we used a second modeling approach to test the five hypotheses proposed to explain the cause of the elevational gradient in species richness. We examined quadratic terms for all variables included in the model except latitude, which was a sixth explanatory variable that we included to account for variation in species richness due to latitude, for a total of 11 potential predictors (Table 1). Each of the five explanatory variables (and their quadratic forms) corresponded to a specific hypothesis proposed to explain elevational variation in avian species richness, after accounting for latitude, and was standardized (scaled) for analysis. We used LASSO regression models with a Poisson error distribution to analyze the relationship between avian species richness and the 11 climatic and geographic explanatory variables (Venables & Ripley, 2002; Friedman, Hastie, & Tibshirani, 2010; R Core Team, 2018). LASSO models are an alternative regularized version of least‐squares regression that includes a penalty for inclusion of each additional explanatory variable to reduce over‐fitting and increase predictive ability and uses cross‐validation to select the model that best fits the data. We divided our data into training (75%) and testing (25%) datasets and used the training data to build, and the testing data to validate, our predictive models. We tested the predictive ability of the candidate models by calculating the correlation between species richness predicted by the model and observed species richness in the 25% testing dataset. We created mountain range‐specific models of species richness, as well as a model that grouped the five western mountain ranges into a single model. We created the later model because of the similarities among the five western mountain ranges in their relationship between elevation and species richness. We combined the data from the five western mountain ranges into a single analysis because it allowed us to increase sample size and statistical power, but we also examined each mountain range separately. Finally, we plotted the relationship between species richness and each of the explanatory variables that were retained in each of the top models selected through cross‐validation (Wickham, 2007; Wickham, 2016; R Core Team, 2018). We created partial effects plots of the relationship between species richness and each explanatory variable in the best‐fit model individually, while holding the remaining variables in the model at their mean values.

**TABLE 1:** Regression coefficients of explanatory variables that helped explain elevational variation in avian species richness for each of the six largest mountain ranges in the United States

Hypothesis	Predictor	Mountain Range						
		Appalachian (*r* = 0.93)	Cascade (*r* = 0.96)	Coast (*r* = 0.87)	Northern Rocky (*r* = 0.90)	Southern Rocky (*r* = 0.97)	Sierra Nevada (*r =* 0.92)	5 Western Mountain Ranges (*r* = 0.91)
N/A	Latitude	0.04	0.001	−0.02	0.003	0.01	0.04	0.01
Area	Area	0.01		0.01	0.02		0.04	0.04
	Area^2^		0.008	−0.01		−0.01	−0.03	−0.02
Productivity	EVI		0.35	0.13	0.18	0.01	0.11	0.17
	EVI^2^	0.05	−0.25	−0.11	−0.12		−0.10	−0.22
Habitat Heterogeneity	Land Cover	0.26	0.73	0.32	0.39	0.44	0.34	0.47
	Land Cover^2^	−0.13	−0.44	−0.19	−0.28	−0.16	−0.20	−0.28
Temperature	Mean Minimum Temp.		0.17		0.12	0.36	0.31	0.31
	Mean Minimum Temp.^2^	0.02	−0.29	−0.04	−0.08	−0.17	−0.26	−0.23
Precipitation	Mean Precipitation	−0.03		−0.02	0.004			
	Mean Precipitation^2^		−0.03			−0.06	−0.05	−0.02

## 3 RESULTS

### 3.1 Species richness gradient

**FIGURE 2:**
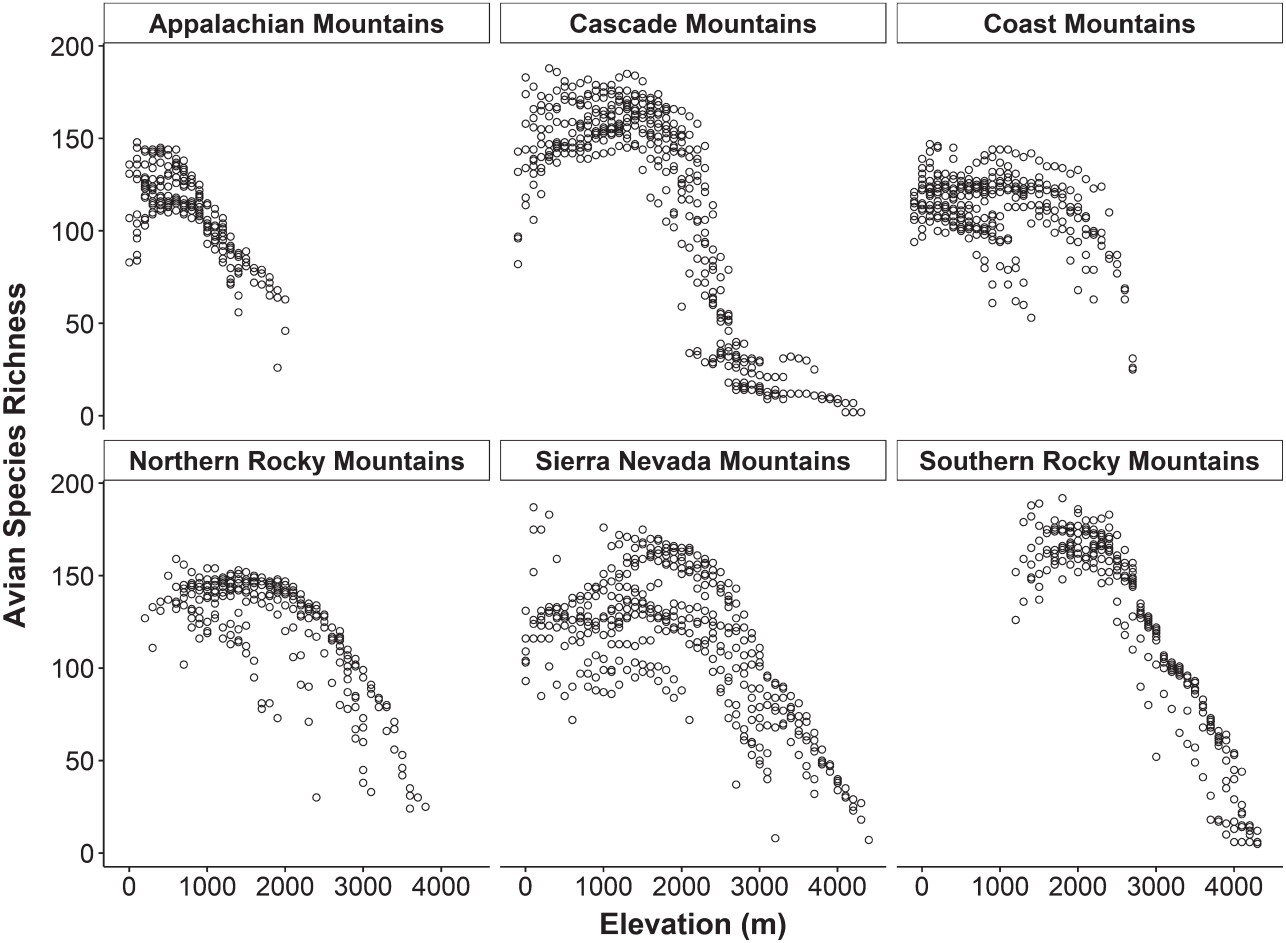
Observed avian breeding season species richness across elevational gradients in six mountain ranges of the United States

We calculated avian species richness within 1,949 sample units comprising 86 elevational transects (12‐18 transects within each of six mountain ranges). The number of transects varied among mountain ranges based on the number of degrees of latitude that the mountain range spanned. Elevational patterns in avian species richness were very similar in all six mountain ranges: it initially increased as elevation increased until a mid‐elevation peak and then decreased at higher elevations (Fig. 2). The raw data suggest that the mid‐elevation peak in species richness occurred at approximately 1500 to 2000 m.a.s.l. in five of the six mountain ranges. The only exception was the one mountain range in eastern North America (the Appalachian Mountains), where the peak occurred at approximately 750 m.a.s.l. Due to this difference, we analyzed data from the Appalachian Mountains separately from the five western mountain ranges in all subsequent analyses. For the five western mountain ranges, we analyzed each separately and also analyzed them pooled together. Pooled results are presented below for brevity due to similarity of relationships observed among the five western mountain ranges that suggested biologically similar patterns.

**FIGURE 3:**
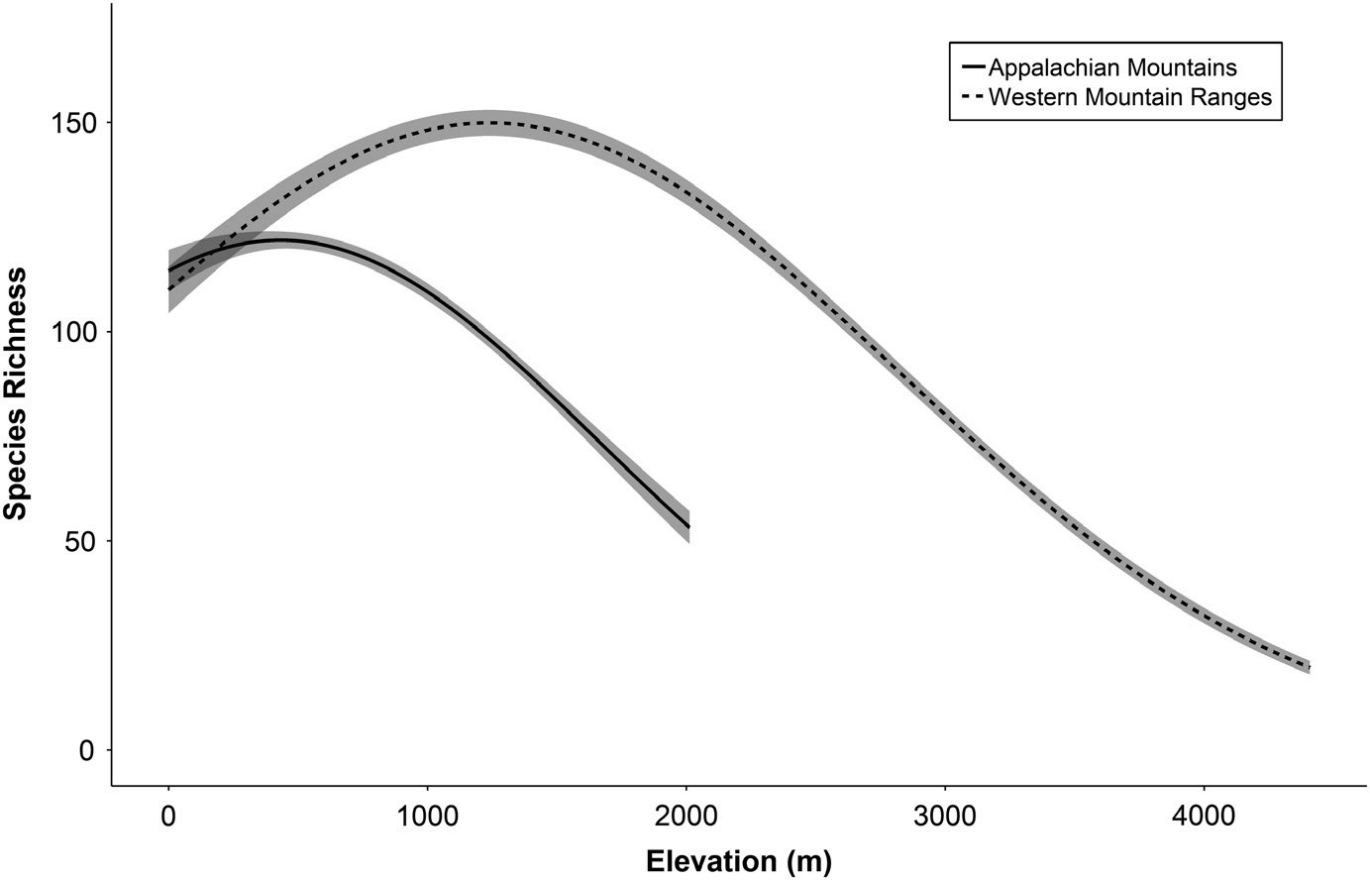
Modeled relationship between avian species richness and elevation in the Cascade, Coast, Sierra Nevada, Northern Rocky, and Southern Rocky Mountains (i.e., the five “western mountain ranges”) and the Appalachian Mountains from a negative binomial regression of species richness on elevation, elevation^2^, and latitude (the informed null model). Latitude was also correlated with species richness and was held at its mean value when creating the graphs. The solid line represents predicted values of species richness and the shaded area represents the 95% confidence interval

Elevation, elevation^2^, and latitude were all statistically significant (p < 0.001) in a negative binomial regression on avian species richness for both models: one with the five western mountain ranges and the one for the eastern mountain range: the Appalachian Mountains (Table 2). In the five western mountain ranges, the model predicted a positive correlation between avian species richness and elevation after controlling for latitude, until species richness peaked at approximately 150 species between 1000 and 1500 m.a.s.l., above which species richness declined (Fig. 3). The model combined the data from the five mountain ranges and predicted a single species richness curve, and that curve suggested a peak in species richness at 1000‐1500 m.a.s.l., slightly lower than the peak observed for the mountain range‐specific raw data (1500‐2000 m.a.s.l). In the Appalachian Mountains, the model also predicted an initial positive correlation between species richness and elevation until species richness reached its peak at approximately 120 species between 500 and 750 m.a.s.l., above which species richness declined (Fig. 3). Both models predicted that species richness was lowest at the highest elevations (Fig. 3; Appendix 1): 15 species for the five western mountain ranges (at 4000 to 4400 m.a.s.l.) and 35 species for the Appalachian Mountains (at 2000 m.a.s.l.). Note that species richness maps in the Appendix were symbolized by “binning” species into groups of 10 for ease of interpretation, occasionally resulting in a blocky appearance of the coloration. The imperfections in coloration were considered less important than the overall interpretability of the maps.

**TABLE 2:** Relationship between avian species richness and elevation and latitude in the Appalachian Mountains and five mountain ranges in the western United States

	Appalachian Mountains			
	Estimate	Standard Error	z	*p*
Elevation	0.0003	0.00005	5.30	<0.001
Elevation^2^	−0.0000003	0.00000003	−11.32	<0.001
Latitude	−0.03	0.003	9.61	<0.001
	**Five Western Mountain Ranges**			
Elevation	0.0005	0.00003	17.88	<0.001
Elevation^2^	−0.0000002	0.000000007	−29.47	<0.001
Latitude	−0.02	0.002	−9.00	<0.001

### 3.2 Predictors of species richness

**FIGURE 4:**
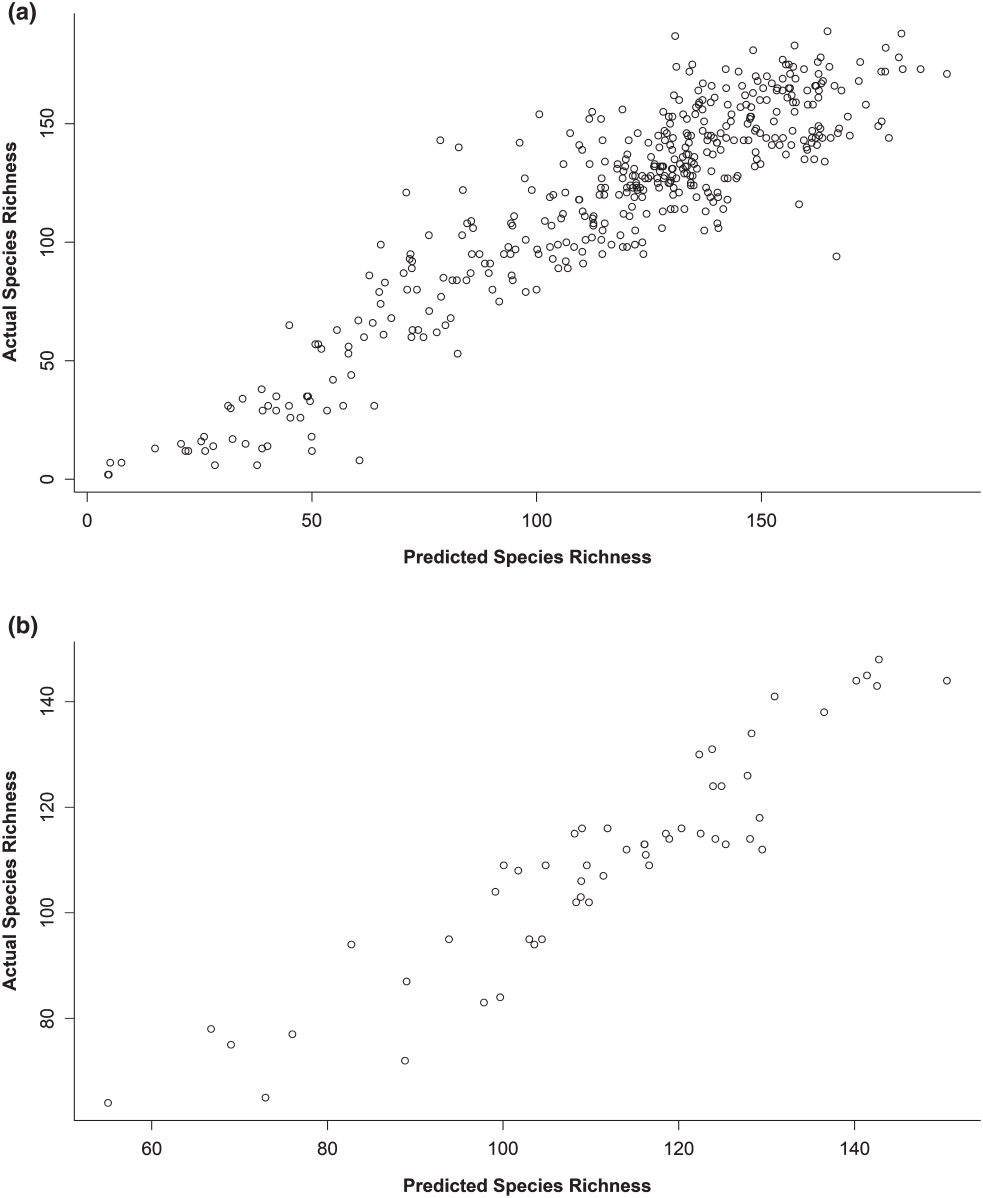
The correlation between the values of species richness predicted by a LASSO regularized regression model and the observed species richness in the testing datasets (25% of the original data, withheld prior to model building). Correlations were high in both the five western mountain ranges (a) and the Appalachian Mountains (b)

Nine of the 10 explanatory variables (all except mean precipitation) helped explain elevational variation in species richness in the five western mountain ranges. The observed values of species richness in the independent testing data were highly correlated (*r*=0.91) with the values predicted by the model (Table 1, Fig. 4a), indicating that the model did a good job of explaining spatial variation in species richness. The model for the Appalachian Mountains also performed well: the species richness values from the independent testing dataset were highly correlated (*r*=0.93) with the values predicted by the model (Table 1, Fig. 4b). Six of the 10 explanatory variables helped explain elevational variation in species richness in the Appalachian Mountains: area, EVI^2^, land cover, land cover^2^, mean of daily minimum temperature^2^, and mean precipitation. All six of the potential explanatory variables in either the linear or quadratic form, or both, were included in every mountain range‐specific model (i.e., all five hypotheses received some support based on the statistical results). Moreover, the correlation between observed and predicted species richness was ≥ 0.90 in all six mountain ranges (Table 1). The correlation between observed and predicted species richness of the informed null model (with only elevation and elevation^2^ included as explanatory variables) was 0.71 in the five combined western mountain ranges and 0.82 in the Appalachian Mountains. That is, the explanatory variables in our mechanistic models (based on the five hypotheses) did a substantially better job of predicting spatial variation in species richness than elevation alone.

**FIGURE 5:**
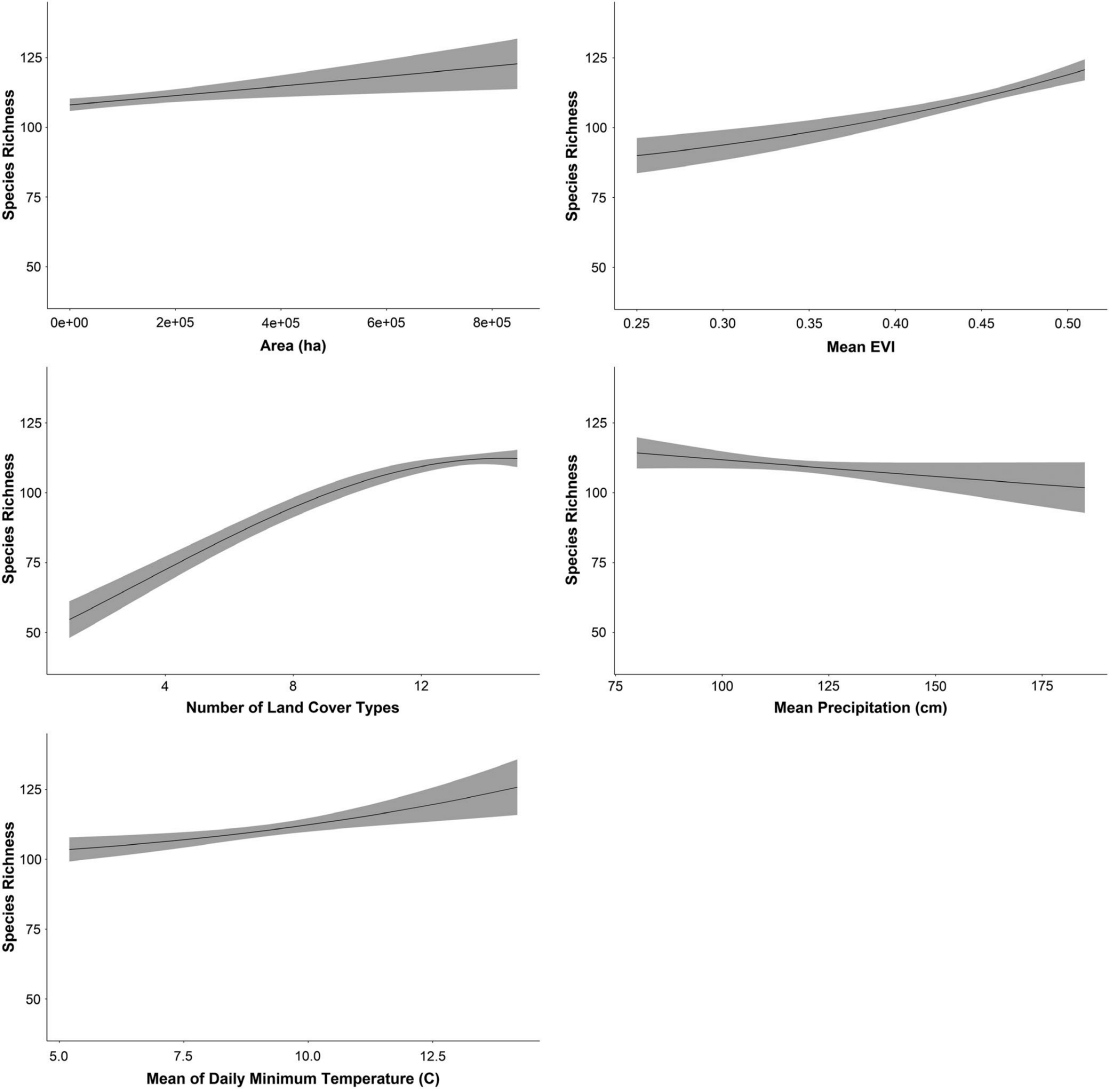
The predicted relationship between each explanatory variable retained in the model and avian species richness, based on a Poisson LASSO model for the Appalachian Mountains

**FIGURE 6:**
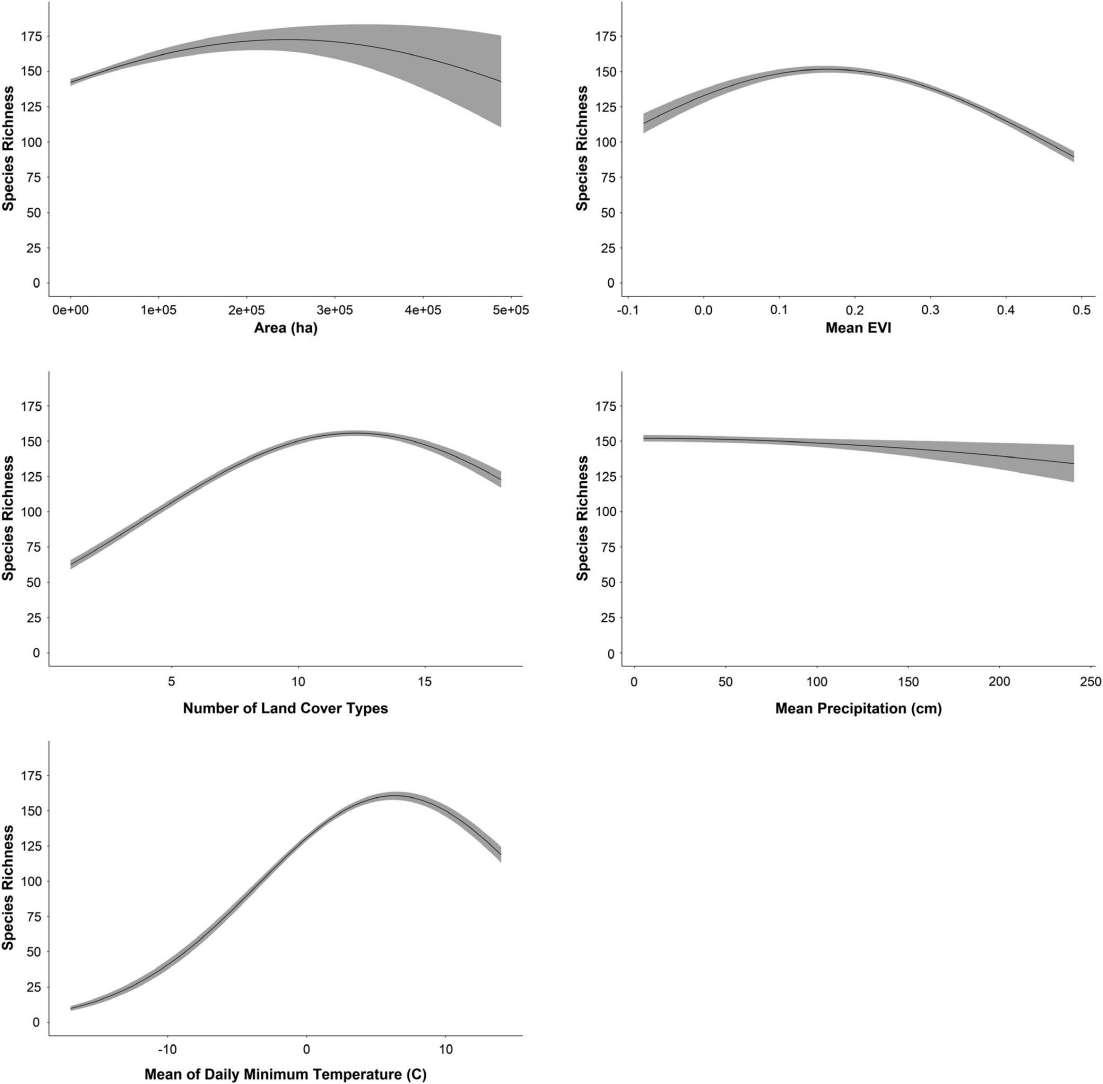
The predicted relationship between each explanatory variable retained in the model and avian species richness, based on a Poisson LASSO model for all five western mountain ranges. Plots for the individual mountain ranges were similar (Figure 7)

**FIGURE 7:**
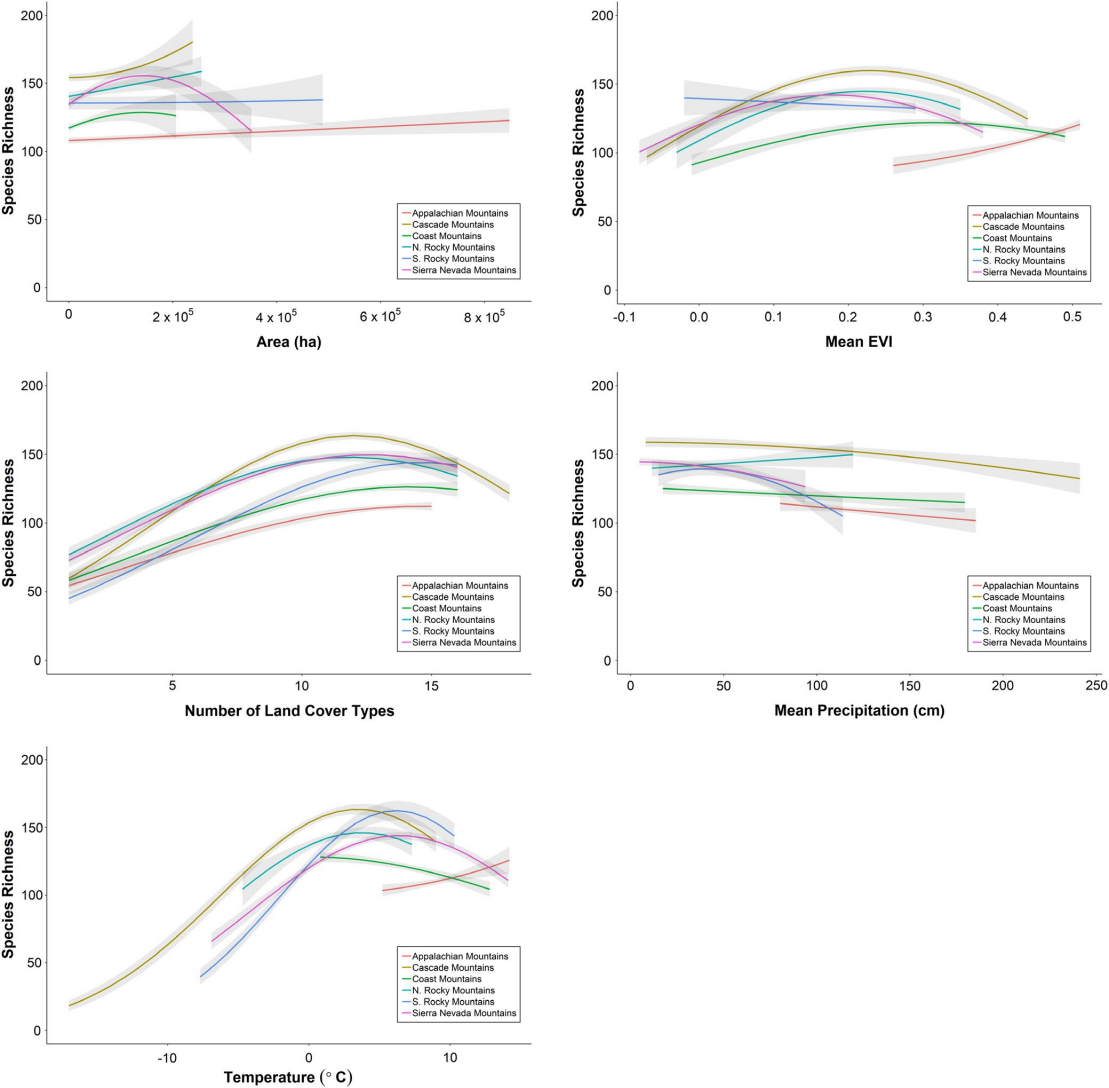
The predicted relationship between avian species richness and habitat heterogeneity (a), temperature (b), EVI (c), area (d), and precipitation (e) for the six largest mountain ranges in the United States, based on a Poisson LASSO model

By examining the shape of the relationship between species richness and each explanatory variable, we inferred the relative strength of support for the five mechanistic hypotheses commonly proposed to explain elevational gradients in species richness. Species richness was positively correlated with both land cover heterogeneity and EVI in the Appalachian Mountains. Species richness was only weakly associated with all other explanatory variables in the Appalachian Mountains (Fig. 5). Species richness was also positively associated with land cover heterogeneity and minimum temperature in the five western mountain ranges and exhibited a unimodal relationship with EVI. Species richness was positively associated with mean minimum temperature from approximately ‐15^◦^ Celsius to 5^◦^ Celsius in the five western mountain ranges, and then plateaued and declined slightly as minimum temperatures increased above 5^◦^ Celsius. Species richness was only weakly associated to all other variables in the five western mountain ranges (Fig. 6). The patterns observed in the five mountain ranges combined were representative of the mountain range‐specific results: a strong positive relationship between species richness and both land cover heterogeneity and minimum temperature was evident in all five mountain ranges, and comparably weaker relationships with all other explanatory variables (Fig. 7).

## 4 DISCUSSION

### 4.1 Species richness gradient

Our results show support for a single, ubiquitous biogeographic pattern throughout the conterminous United States whereby species richness of breeding birds varies along elevational gradients in a similar nonlinear pattern, with medium levels of richness at the lowest elevations, increases in richness to a mid‐elevation peak, and declines in richness with further increases in elevation, with the lowest species richness at the highest elevations (Fig. 1c). This general pattern has been previously reported in studies of elevational variation in species richness in birds, reptiles, and small mammals and has been referred to as a *low elevation plateau with a mid‐elevation peak* (McCain, 2009; McCain & Grytnes, 2010). In contrast, our results were not consistent with a parabolic pattern of species richness that would be predicted by the mid‐domain effect (Fig. 1d; Colwell & Lees, 2000). Additionally, our results refute the historical assumption that species richness uniformly declines with increasing elevation (MacArthur, 1972; Stevens, 1992) and corroborates results from more recent studies that have also documented a mid‐elevation peak as one of the most commonly observed elevational patterns of species richness in birds (McCain, 2009). Our results are unique in that we documented the same general relationship between species richness and elevation in all six of the largest mountain ranges of the United States, despite substantial differences among the six mountain ranges in major geographic characteristics such as range insularity, vegetative communities, topography, climate, and geology. The primary difference we observed among the six mountain ranges was the threshold elevation above which species richness began to decline. In all five western mountain ranges—the Cascade, Coast, Sierra Nevada, Northern Rocky, and Southern Rocky Mountains—the nonlinear elevational pattern in avian species richness was strikingly similar: a peak at approximately 1500 to 2000 m.a.s.l. and a sharp decline above that elevation. Species richness exhibited the same overall elevational pattern in the Appalachian Mountains, but the peak and, hence, the sharp decline began at a much lower elevation (750 m.a.s.l.). Further research is needed to explore why these differences occur, but the Appalachian Mountains have a much lower peak elevation overall and may experience more human disturbance at higher elevations than the five western mountain ranges included in this study (Lepczyk et al., 2008).

### 4.2 Predictors of species richness

We found strong support for two well‐known hypotheses (the habitat heterogeneity and temperature hypotheses), as well as some support for the primary productivity hypothesis, as an explanation for the nonlinear relationship between elevation and avian species richness in the United States. The habitat heterogeneity hypothesis suggests that species richness is positively related to habitat heterogeneity, such that a higher diversity of vegetation types should support a larger number of species (Lack, 1969; Dunning, Danielson, & Pulliam, 1992). Indeed, previous studies have found that land cover heterogeneity is positively correlated with avian species richness (Rittenhouse et al., 2012; Morelli et al., 2013). From a mechanistic perspective, increased habitat heterogeneity is indicative of diverse food resources, foraging habitat availability, breeding habitat availability, nesting sites, and thermoregulatory demands. That is, environmental heterogeneity is hypothesized to increase niche availability for species with diverse resource and physiological needs (Bazzaz, 1975; Pigot, Trisos, & Tobias, 2016). Thus, a wider variety of landcover types can support birds with a wider variety of niche requirements and, hence, species richness is predicted to increase along a gradient of increasing habitat heterogeneity. The scale at which past studies have measured habitat heterogeneity has varied from local‐scale heterogeneity (Freemark & Merriam, 1986; Goetz et al., 2007) to heterogeneity of habitat types (Bohning‐Gaese, 1997; Stein, Gerstner, & Kreft, 2014; this study) and the mechanism likely operates at both of these scales.

Temperature may constrain species richness by increasing thermoregulatory demands beyond some species’ ability to cope or by limiting foraging opportunities, effectively imposing a physiological limit on range size (Janzen, 1967). Indeed, bird distributions have been shown to be limited by thermoregulatory costs and adaptations (Lennon, Greenwood, & Turner, 2001; Londoño, Chappell, Jankowski, & Robinson, 2017), including local‐scale distributions of birds along elevational gradients (Boyle et al., 2010). Thus, species richness is predicted to be positively correlated with temperature (Currie, 1991; McCain, 2007). Primary productivity is thought to be strongly correlated with food resource abundance for vertebrate and invertebrate taxa (Loeb, Siegel, Holm‐Hansen, Hewitt, Fraser, et al., 1997; Hurlbert & Haskell, 2003). Therefore, species richness is predicted to be positively correlated with primary productivity, due to the increased abundance of food resources (Connell & Orias, 1964; Wright, 1983). Indeed, bird diversity has previously been shown to increase along primary productivity gradients (Hurlbert & Haskell, 2003; Bailey et al., 2004).

The hypotheses found to best explain the observed pattern, when taken together, suggest evidence for a unifying mechanistic explanation for elevational gradients in avian species richness in North America. All three of the most strongly supported hypotheses—Habitat Heterogeneity, Temperature, and Primary Productivity—are associated directly or indirectly with resource availability. Higher habitat heterogeneity is associated with increased structural diversity, which provides foraging and nesting opportunities for bird species with a variety of niche requirements (Willson, 1974; Martin, 1988b). Indeed, several previous studies reported a strong relationship between species richness and habitat heterogeneity (Hurlbert, 2004; Stein, Gerstner, & Kreft, 2014; Yang et al., 2015). Our results corroborate these studies and provide the most thorough and geographically rigorous demonstration of a positive or unimodal relationship between species richness and habitat heterogeneity. Likewise, primary productivity is commonly considered an indicator of avian food availability (Pettorelli et al., 2005). Temperature could influence species richness directly through physiological constraints or indirectly via food availability (Hawkins et al., 2003). Hence, we propose that elevational variation in food availability provides the mechanistic link among these three hypotheses and explains why all three are strong predictors of elevational variation in avian species richness, in addition to the direct impacts of each process in isolation.

In this study, we used a novel approach to investigate the ecological drivers of elevational gradients in bird diversity by using high‐resolution avian distribution maps to calculate species richness. These maps are not “smoothed” across landscapes and elevations, as a typical distribution map often is, but are instead sensitive to fine‐scale variation in habitat suitability. One of the advantages of this approach is that the species occurrence data was not subject to the limitations of time, scale, and observer bias often inherent in obtaining raw survey data over large areas. Moreover, the collection of survey data across elevation gradients in particular is often inhibited by topography in terms of both access and detection probability. Nevertheless, all datasets have limitations. Although, the 30m‐resolution species distribution maps we used were from habitat models based on literature reviews and incorporated a broad range of topographical, vegetative, habitat, and other ecological variables, a model can never be a perfect depiction of reality. Future investigations might compare the results from raw survey data and the species distribution models used here within a single study, and ground‐truth model predictions.

Will species adapt or shift their distributions in response to climate change? The answer to this question will determine the effects of future climate change scenarios on species diversity. Assisted migration (Peters & Darling, 1985, McLachlan, Hellman, & Schwartz, 2007, Hewitt et al., 2011) has been proposed as a hands‐on approach to help plants and animals shift their distributions and thereby prevent extinctions caused by climate change. But such efforts assume that climate (and temperature in particular) is the only, or at least the primary, process that limits species distributions and, hence, the primary cause of gradients in species diversity along elevational and latitudinal gradients. Our results provide compelling evidence that abiotic factors do explain some of the variation in avian species richness along elevational gradients, but other processes also influence contemporaneous gradients in species richness and the relative importance of temperature likely differs across taxa.

## CONFLICT OF INTEREST

The authors have no conflicts of interest to declare.

## Author Contribution

**Kristen G. Dillon:** Conceptualization (lead); Data curation (lead); Formal analysis (lead); Funding acquisition (equal); Investigation (lead); Methodology (lead); Visualization (lead); Writing‐original draft (lead); Writing‐review & editing (equal). **Courtney J. Conway:** Conceptualization (supporting); Data curation (supporting); Formal analysis (supporting); Funding acquisition (equal); Investigation (supporting); Methodology (supporting); Writing‐original draft (supporting); Writing‐review & editing (equal).

## ETHICAL STATEMENT

No live animals or specimens were used in this research.

## Supporting information

Supplementary Material

## Data Availability

Data are available via the Dryad Digital Repository: https://doi.org/10.5061/dryad.612jm642z
