# Supplementary material for: Habitat heterogeneity, temperature, and primary productivity drive elevational gradients in avian species diversity
Source: Ecol Evol. 2021 May 1;11(11):5985–97. doi: 10.1002/ece3.7341 (PMC8207161; doi:10.1002/ece3.7341)

S1. Observed avian species richness and elevation gradients in the Appalachian Mountains.

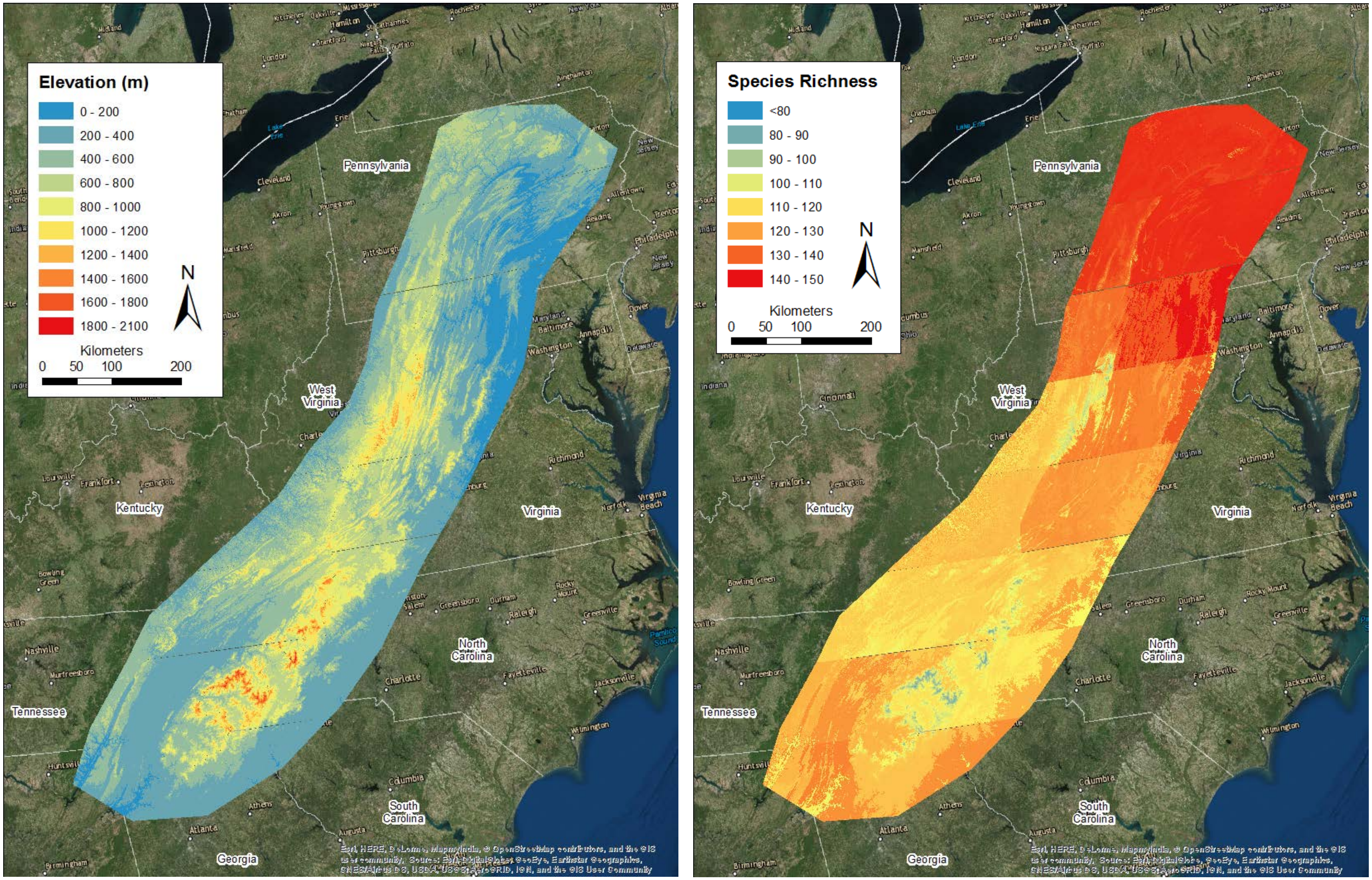

S2. Observed avian species richness and elevation gradients in the Cascade Mountains.

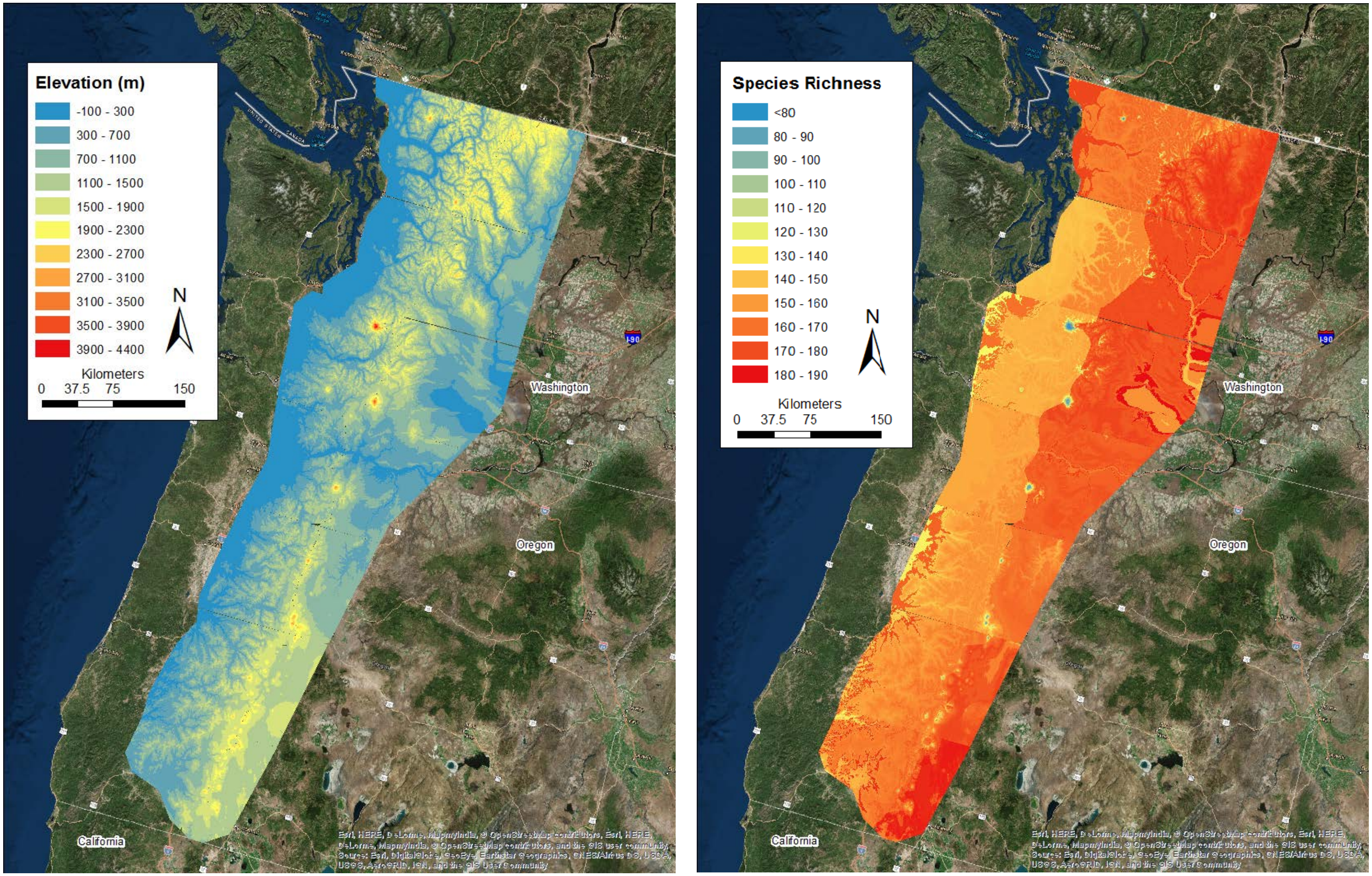

S3. Observed avian species richness and elevation gradients in the Coast Mountains.

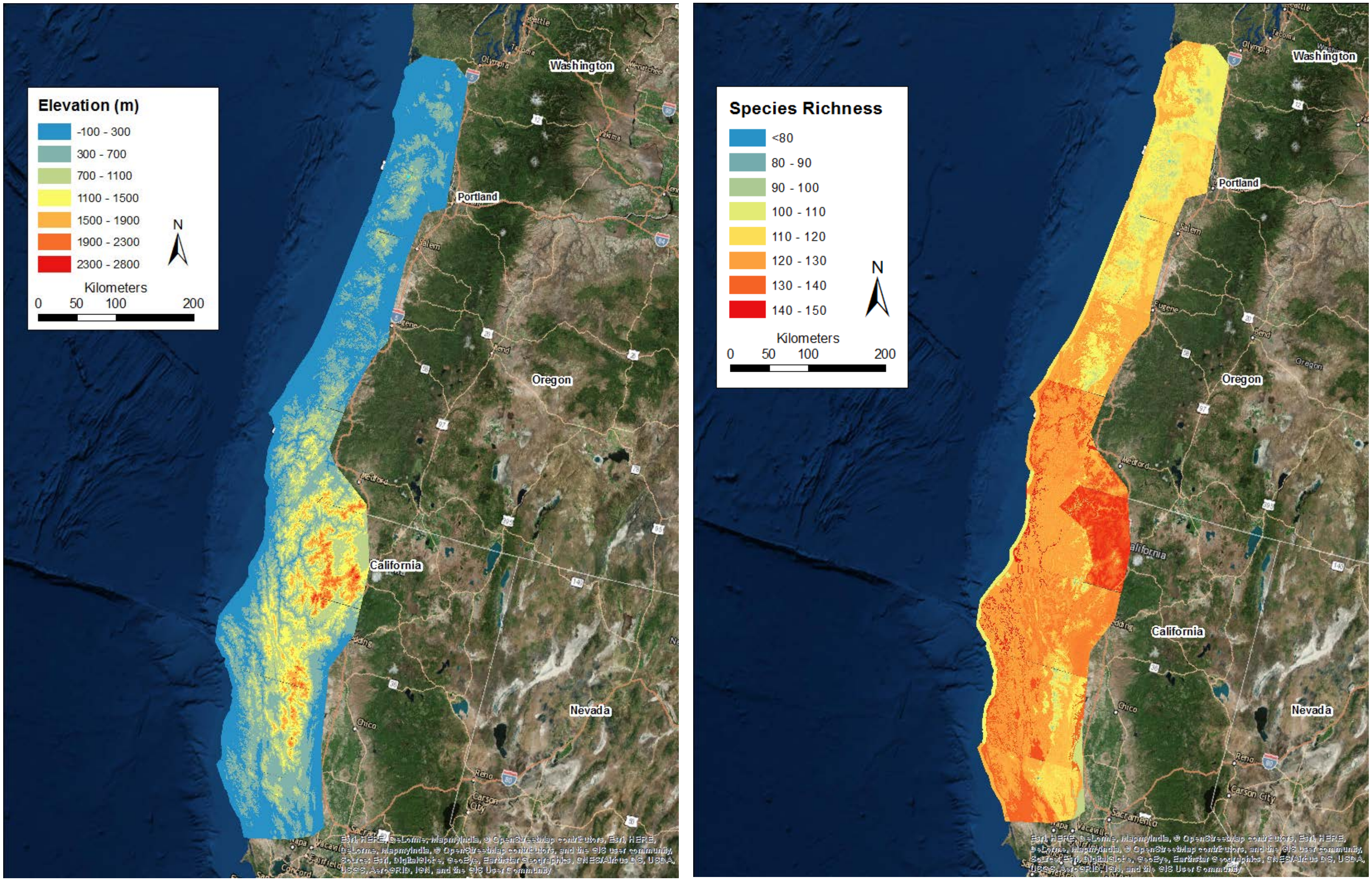

S4. Observed avian species richness and elevation gradients in the Sierra Nevada Mountains.

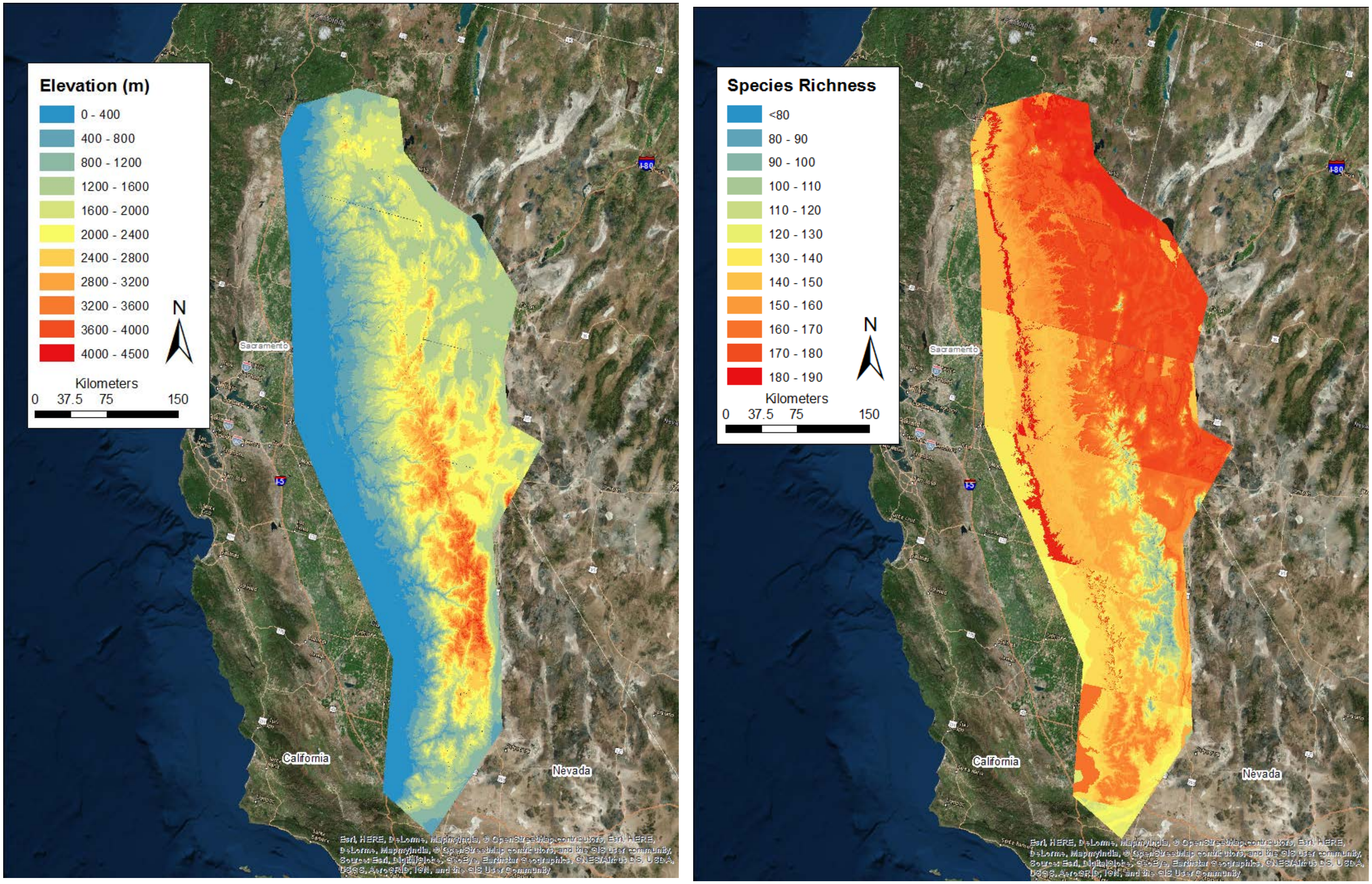

S5. Observed avian species richness and elevation gradients in the Northern Rocky Mountains.

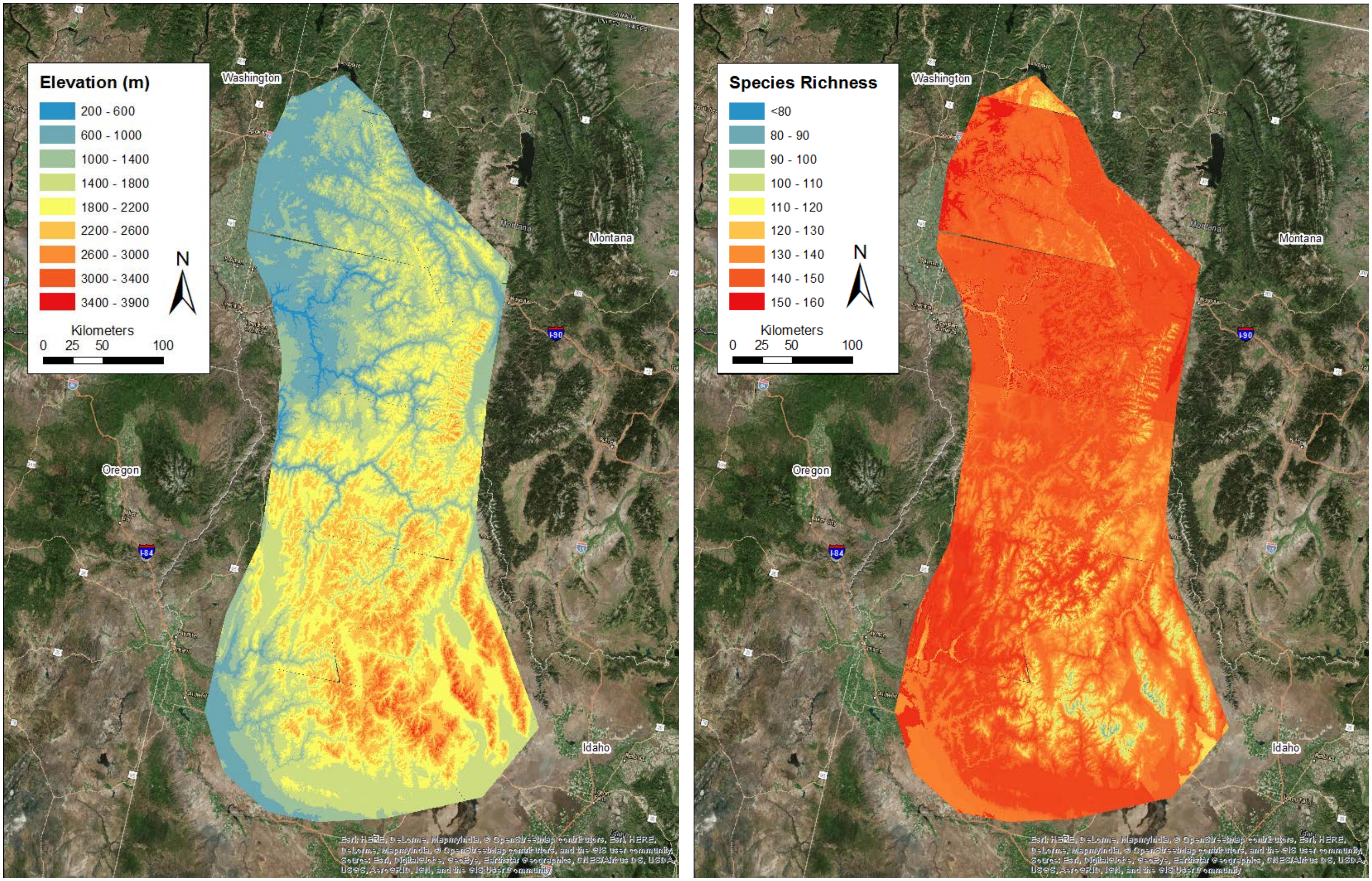

S6. Observed avian species richness and elevation gradients in the Southern Rocky Mountains.

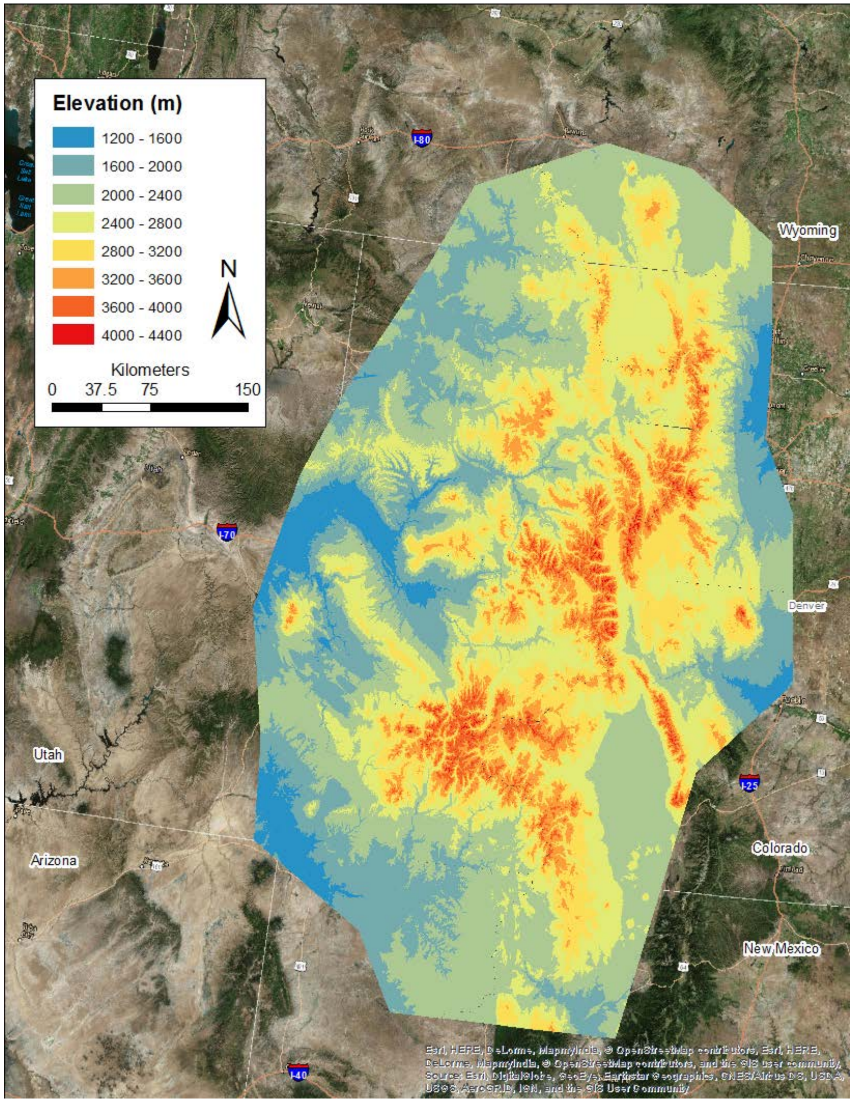

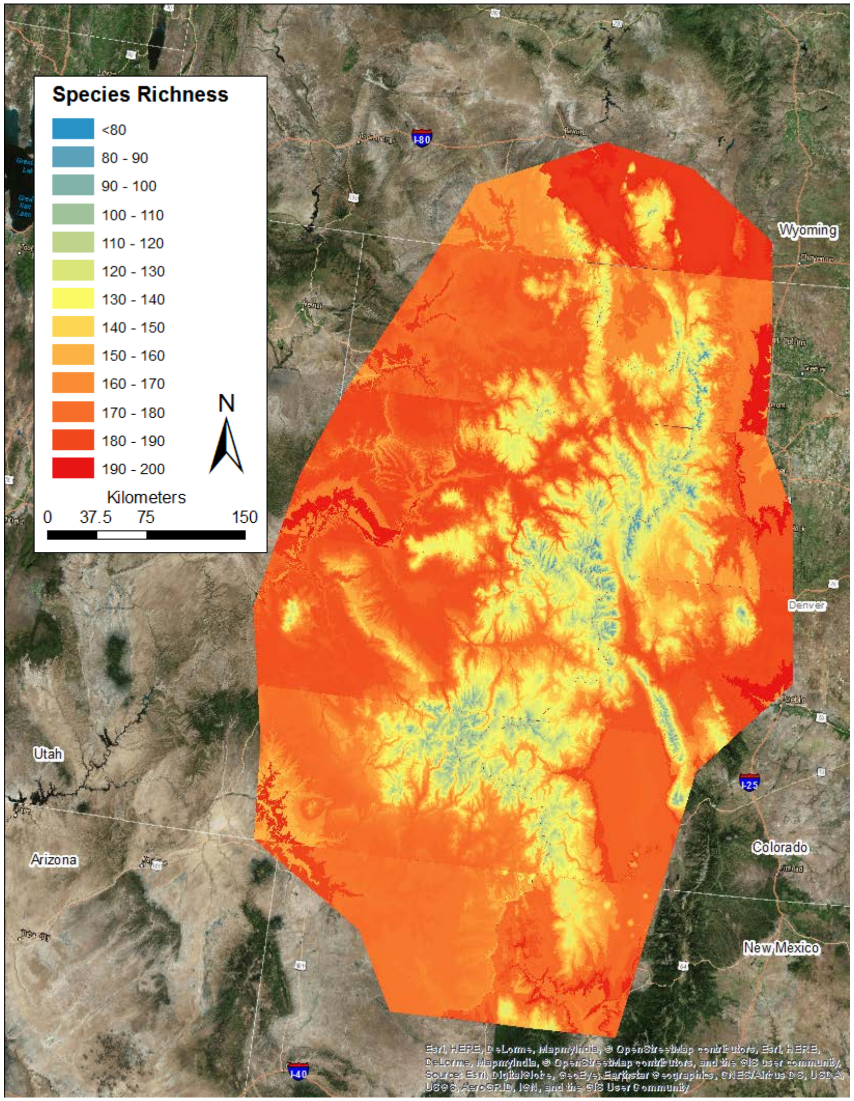

Supplement: Supplementary file 1 — Supplementary Material [file ECE3-11-5985-s001.pdf]
